# Optical coherence tomography angiography biomarkers in multiple sclerosis and neuromyelitis optica spectrum disorders: a systematic review

**DOI:** 10.1186/s40942-025-00698-x

**Published:** 2025-06-23

**Authors:** Omid Mirmosayyeb, Mohammad Yazdan Panah, Reza Kord, Elahe Espoo, Saeed Vaheb, Aram Zabeti, Vahid Shaygannejad

**Affiliations:** 1 Isfahan Neurosciences Research Center, Isfahan University of Medical Sciences, Isfahan, Iran; 2 Department of Neurology, Isfahan University of Medical Sciences, Isfahan, Iran; 3 Department of Neurology, University of Cincinnati, Cincinnati, OH USA

**Keywords:** Optical coherence tomography angiography, Multiple sclerosis, Neuromyelitis optica spectrum disorder

## Abstract

**Background:**

Multiple sclerosis (MS) and neuromyelitis optica spectrum disorder (NMOSD) are autoimmune disorders of the central nervous system with overlapping clinical manifestations but distinct treatments and prognoses. Imaging markers are necessary to differentiate between these disorders, especially when serologic testing is unavailable or unclear. Optical coherence tomography angiography (OCT-A) serves as a non-invasive imaging tool that assesses retinal microvascular alterations, potentially as a modality for differentiating MS and NMOSD. This review aimed to assess and consolidate evidence on retinal vascular alterations, measured by OCT-A, in people with MS (PwMS) and people with NMOSD (PwNMOSD) to help discriminate between these disorders.

**Methods:**

PubMed/MEDLINE, Web of Science, Scopus, and Embase were systematically searched up to August 27, 2024, to identify original English studies that compared OCT-A parameters between PwMS and PwNMOSD. The risk of bias across studies was evaluated utilizing the Newcastle–Ottawa Scale (NOS). Findings were consolidated using a narrative synthesis method.

**Results:**

Nine studies involving 181 PwMS and 166 PwNMOSD were included. Compared to PwMS, PwNMOSD exhibited significantly lower vessel densities in the peripapillary and macular regions, reduced radial peripapillary capillary (RPC) density, and smaller foveal avascular zone (FAZ) areas, particularly in optic neuritis (ON)-affected eyes. Minimal differences were observed in eyes without ON, suggesting that ON may be crucial when utilizing OCT-A biomarkers for disease discrimination.

**Conclusion:**

OCT-A metrics demonstrate potential as biomarkers that may help distinguish MS and NMOSD, with PwNMOSD showing more severe retinal vascular alterations. These preliminary findings highlight that OCT-A may hold promise as a diagnostic tool for differentiating MS and NMOSD. Further studies are needed to validate these findings.

**Supplementary Information:**

The online version contains supplementary material available at 10.1186/s40942-025-00698-x.

## Introduction

Multiple sclerosis (MS) is a chronic autoimmune disorder marked by inflammatory processes, demyelination, and axonal degeneration in the central nervous system (CNS) [1]. Common presenting symptoms of multiple sclerosis (MS) include acute optic neuritis (ON), partial myelitis, and brainstem symptoms; it primarily affects young and female adults and poses significant socioeconomic challenges globally [2]. Incorporating differential diagnoses such as neuromyelitis optica spectrum disorder (NMOSD) into the diagnostic evaluation of MS is crucial, as NMOSD can present with similar symptoms and clinical manifestations while requiring distinct therapeutic strategies and resulting in different prognostic outcomes [3, 4].

NMOSD is classified as an autoimmune disorder of the CNS that primarily involves the optic nerve and spinal cord [5]. The pathogenic mechanisms, in most cases, are characterized by astrocytic damage mediated by autoantibodies targeting aquaporin-4 (AQP4-ab) [5]. Optic neuritis (ON) serves as a principal manifestation of NMOSD, resulting in visual impairment due to extensive neuroaxonal damage affecting the optic nerve and the retina [6]. Although AQP4-ab is frequently detected in a substantial proportion of PwNMOSD, there remains a critical need for additional data to substantiate clinical and neuroimaging observations, particularly in individuals who are negative for AQP4-ab [7].

The distinction between MS and NMOSD is critical due to their overlapping clinical and radiological characteristics [8]. There are significant differences in the treatment approaches for people with MS (PwMS) and people with NMOSD (PwNMOSD), and inadequate treatment can lead to repeated disease episodes that accelerate neurological function decline [9]. Furthermore, therapeutic interventions for MS may inadvertently exacerbate the clinical course of NMOSD [10]. Currently, the differential diagnosis relies primarily on the detection of AQP4-ab; however, around 25% of patients may be seronegative or possess anti-MOG antibodies (MOGAD) [9]. Thus, it seems that distinguishing between MS and NMOSD is still challenging [11].

Recent studies have increasingly focused on the vascular alterations linked to MS and its differential diagnoses, such as NMOSD [3]. Recent investigations have demonstrated that retinal vessel loss occurs in PwMS [12, 13] and may also be present in PwNMOSD [14]. While ON is a prevalent manifestation in both conditions, PwNMOSD frequently experience bilateral optic nerve involvement, which is associated with poorer visual outcomes [15]. Optical coherence tomography (OCT) is a crucial diagnostic tool for MS by identifying thinning in the retinal nerve fiber layer (RNFL) and the retinal ganglion cell layer (GCL) [16]. Recently, optical coherence tomography angiography (OCT-A) has emerged as an advanced imaging technique providing non-invasive, depth-resolved visualization of vascular networks and flow velocity in the optic disc and macula [17, 18]. OCT-A can be utilized in various innovative approaches to enhance the comprehension of neuroinflammatory diseases [19].

Preliminary findings from OCT-A studies suggest that the retinal microvasculature is compromised in both MS and NMOSD, regardless of the presence of ON, and in some instances, even in the absence of structural retinal atrophy [13, 20]. Although various OCT-A parameters, such as vessel density (VD) [7, 21], foveal avascular zone (FAZ) [7], perfusion density (PD) [21], and retinal capillary plexus (RCP) [22], have been identified as potentially relevant for distinguishing between MS and NMOSD, but a consensus on their implication has yet to be established. To better understand the potential applications of retinal vasculature in differentiating between MS and NMOSD, this systematic review aimed to synthesize and evaluate evidence on disease-specific alterations in the retinal vasculature, as assessed using OCT-A, to help distinguish between MS and NMOSD.

## Methods

### Study design

The current systematic review was conducted in accordance with the PRISMA guidelines [23]. Prior to its execution, this review was registered with the PROSPERO registry under the reference number CRD42024603556. Since all data included in the study are publicly accessible, neither institutional ethical review nor patient consent was necessary.

### Search strategy

A systematic electronic literature search was conducted on August 27, 2024, utilizing the PubMed/MEDLINE, Scopus, Embase, and Web of Science databases, in addition to a manual examination of pertinent references from retrieved published studies. Keywords and medical subject headings (MeSH) pertinent to MS, NMOSD, and OCT-A were employed. An overview of the detailed search methodologies applied across the various databases is included in Supplementary Material, Table S1. To reduce the risk of missing eligible studies, reference lists of included studies were manually reviewed.

### Study selection

All studies identified through the search process and additional sources were imported into an EndNote X21 (Clarivate) database to eliminate duplicates. Two reviewers (RK and ES) screened the titles and abstracts based on predetermined selection criteria. Each study deemed potentially relevant was evaluated independently, and full-text articles were acquired for further assessment regarding eligibility for inclusion. Discrepancies concerning the selection of specific studies were addressed by referring to a third reviewer (OM). The final decision was reached through a group discussion involving all authors.

### Eligibility criteria

Peer-reviewed studies published in English were considered eligible if they fulfilled the following conditions: (a) participants were adults over 18 years of age with a confirmed diagnosis of MS or NMOSD based on the most widely accepted and recent diagnostic criteria: the 2010 and 2017 revised McDonald criteria for MS [24, 25] and the 2015 International Consensus Diagnostic Criteria for NMOS [26], (b) the study design was cross-sectional, case-control, or cohort, and (c) OCT-A parameters were simultaneously assessed in PwMS and PwNMOSD.

### Data extraction

Two reviewers (RK and SV) independently extracted relevant data utilizing a standardized proforma that captured publication details, study design, sample size, demographic and clinical characteristics of participants, outcome measures, specifications of OCT-A devices, and major findings from the studies. When the provided data were insufficient or presented only in graphical form, supplementary information, and clarifications were sought from the principal author of the studies. Discrepancies were addressed through consensus among all reviewers, and all data were thoroughly validated for precision.

### Risk of bias assessment

The risk of bias (ROB) of the included studies was evaluated utilizing the Newcastle–Ottawa Scale (NOS) [27]. Two reviewers (ES and SV) assessed the NOS outcomes for each article, and discrepancies were addressed through discussions with the third reviewer (OM). For case-control studies, three domains were assessed: study selection (adequate case definition and representativeness, as well as appropriate control selection), comparability, and exposure (ascertainment methods, consistency in case and control ascertainment, and non-response rates). Each publication was rated with a total star score from 0 to 3, indicating low quality (high risk of bias), 4 to 6 as moderate quality (moderate risk of bias), and 7 to 9 as high quality (low risk of bias) [28].

### Data synthesis and presentation

The data extraction was organized in tables, with a narrative synthesis used to demonstrate patterns in the findings [29]. This approach identified gaps in the literature regarding the role of OCT-A in distinguishing between MS and NMOSD. Additionally, data from various articles were compared, where relevant, to uncover any underlying trends or influences.

## Results

### Search results

**Fig. 1:**
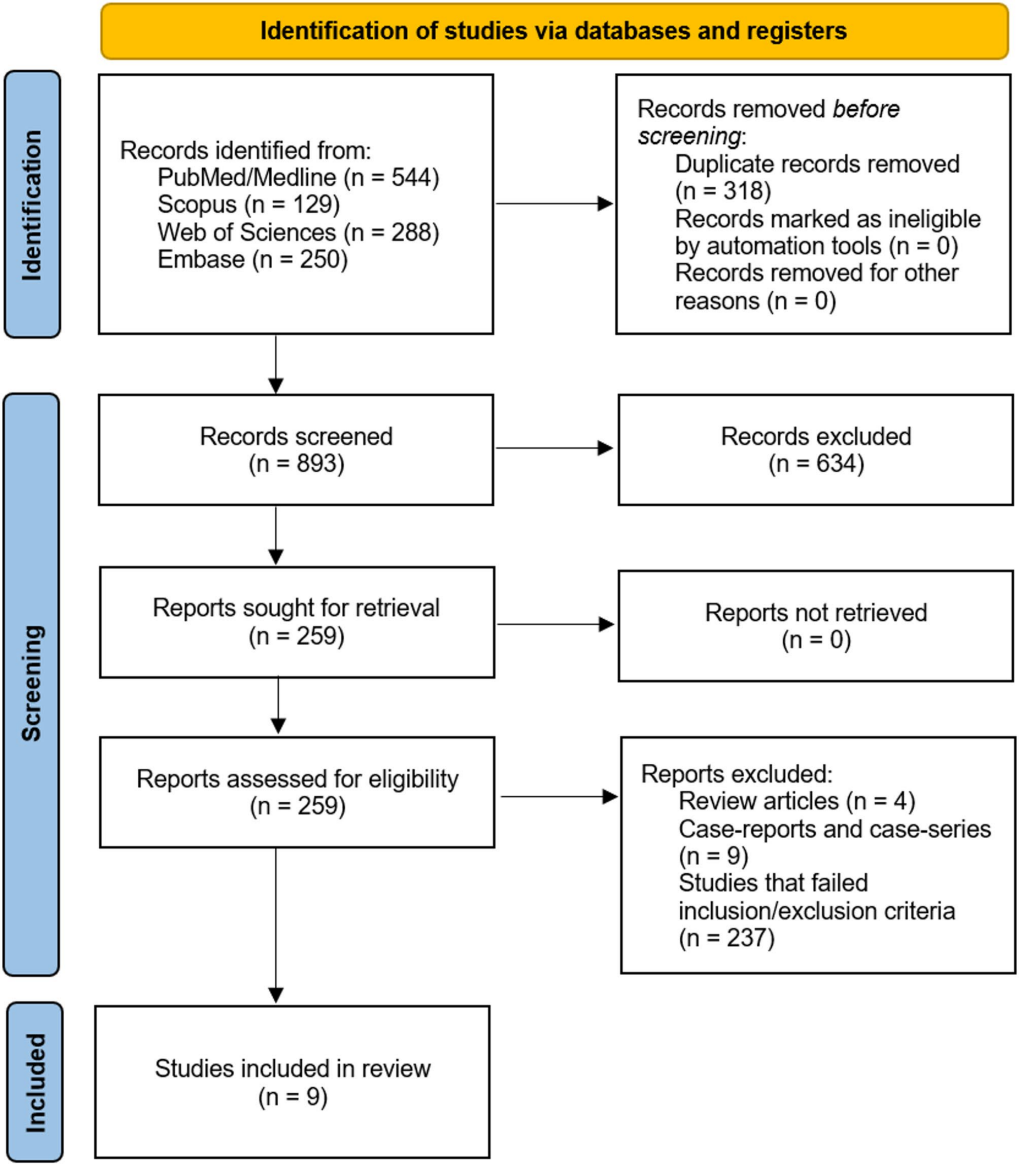
PRISMA flow diagram of searching databases and study selection process

The database search initially identified 1211 articles. Following the removal of 318 duplicates, 893 articles were subjected to a two-stage screening process. First, titles and abstracts were reviewed, resulting in the exclusion of 634 articles. The full texts of the remaining 259 articles were then assessed, leading to the elimination of 250 more. Ultimately, nine studies were selected for inclusion in this systematic review, as illustrated in the PRISMA diagram (Fig. 1).

### The main characteristic of the included studies

Table 1 presented an overview of the principal features of the included studies. All nine studies employed a case-control design, with four conducted in Poland [30–33], two in China [21, 34], one in Germany [35], one in Korea [22], and one in Turkey [7]. Four studies from Poland [30–33] and two studies from China [21, 34] involved the same study populations within their respective countries; however, they were conducted for distinct research objectives. The included studies comprised 181 PwMS (70.7% female) with a mean (SD) age of 33.8 (9.5) years and a disease duration of 10 (5.7) years, as well as 166 PwNMOSD (87.3% female), of whom 115 (89.1%) were AQP4-seropositive, with a mean (SD) age of 42.8 (16.2) years and a disease duration of 5.7 (5) years. All included studies classified PwMS into MS-ON and MS-NON groups and PwNMOSD into NMOSD-ON and NMOSD-NON groups. OCT-A measurements were conducted in five studies using the Optovue RTVue XR Avanti (Optovue Inc., Fremont, CA) with AngioVue software [30–33, 35], while four studies utilized the Zeiss Cirrus (Carl Zeiss Meditec, Inc., Dublin, CA), three of which employed AngioPlex [7, 21, 34] software, and one study used the Topcon OCT-A instrument [22].

**Table 1:** The main characteristics of included studies

Author	Country Year	Study Design	PwMS				PwNMOSD				OCTA Device/ Software	ROI	OCTA Parameters	QA
			MS Type	Sample Size/ F: M/ Age: mean (SD)	EDSS: mean (SD)	Disease Duration: mean (SD)	Sample Size/ F: M/ Age: mean (SD)	EDSS: mean (SD)	Disease Duration: mean (SD)	AQP4-IgG Antibodies				
C. Liu [21]	China 2024	Case-control	NR	83 56:27 31 (27–38)**	2 (1.5–3.2)**	5 (2-9.3)**	91 81:10 41 (29-53.5)**	3 (2–3)**	5 (2.6-9)**	Seropositive:91	Zeiss Angio Plex	Macular	VD PD	8
L. Aly [35]	Germany 2022	Case-control	RRMS:21	21 16:5 38 (11.4)	1.4 (1.2)	5.6 (4.1)	16 13:3 46.6 (10)	3.4 (2.4)	6.1 (2.5)	Seropositive:10 Seronegative:6	Optovue Angio Vue	Macular	SVC DVC FAZ	7
B. Tiftikcioglu [7]	Turkey 2022	Case-control	RRMS:14	14 6:8 27.6 (7.4)	NR	3.3 (3.3)	9 8:1 35.9 (14.9)	NR	5.7 (3.1)	Seropositive:9	Zeiss Angio Plex	Macular Peripapillary	VD FAZ	5
G. lee [22]	Korea 2021	Case-control	NR	23 18:5 38 (13)	NR	2.9 (1.7)	37 32:5 47 (13)	NR	3.7 (1.8)	NR	Zeiss Topcon OCT instrument	Macular Peripapillary	DRCP SRCP	7
C. Liu [34]	China 2021	Case-control	NR	83 56:27 31 (27–38)**	NR	5 (2-9.4)**	91 81:10 41 (29-53.8)**	NR	5 (2.6–8.9)**	Seropositive:91	Zeiss Angio Plex	Macular	PD VD FAZ	8
M. Rogaczewska [30]	Poland 2021	Case-control	NR	40 32:8 35.1 (7.4)	NR	8 (3–32)*	13 11:2 42.1 (10.2)	NR	9 (1–33)*	Seropositive:13	Optovue Angio Vue	Macular Peripapillary	SCP DCP RPC	6
M. Rogaczewska [31]	Poland 2021	Case-control	NR	40 32:8 35.1 (7.4)	NR	8 (3–32)*	13 11:2 42.1 (10.2)	NR	9 (1–33)*	Seropositive:13	Optovue Angio Vue	Macular Peripapillary	RPC DCP SCP	6
M. Rogaczewska [32]	Poland 2021	Case-control	NR	40 32:8 35.1 (7.4)	NR	8 (3–32)*	13 11:2 42.1 (10.2)	NR	9 (1–33)*	Seropositive:13	Optovue Angio Vue	Macular Peripapillary	RPC	6
M. Rogaczewska [33]	Poland 2021	Case-control	NR	40 32:8 35.1 (7.4)	NR	8 (3–32)*	13 11:2 42.1 (10.2)	NR	9 (1–33)*	Seropositive:13	Optovue Angio Vue	Macular Peripapillary	SCP DCP RPC	6

*Median (range), **Median (IQR), ^m^Months, Mean (Range)^α^. AQP4: aquaporin-4, DCP: deep capillary plexus, DRCP: deep retinal capillary plexus, DVC: deep vascular complex, EDSS: expanded disability status scale, FAZ: foveal avascular zone, MS: multiple sclerosis, NMOSD: neuromyelitis optica spectrum disorder, NR: not reported, OCT: optical coherence tomography, OCT-A: optical coherence tomography angiography, PD: peripapillary density, PwMS: people with multiple sclerosis, PwNMOSD: people with neuromyelitis optica spectrum disorder, QA: quality assessment, ROI: region of interest, RPC: radial peripapillary capillaries, RRMS: relapsing-remitting multiple sclerosis, SD: standard deviation, SCP: superficial capillary plexus, SRCP: superficial retinal capillary plexus, SVC: superficial vascular complex, VD: vascular density

### Results of individual studies

Liu et al. carried out a cross-sectional study on 83 PwMS and 91 seropositive PwNMOSD to assess the capacity of OCTA for disease discrimination. The findings indicated that the overall VD and PD areas were greater in PwMS compared to those with NMOSD [21, 34]. Specifically, PwMS exhibited higher macular VD in the inferior-inner, superior-inner, inferior-outer, superior-outer, and nasal-outer quadrants, along with increased macular PD in the superior-outer, inferior-outer, superior-inner, and nasal-outer quadrants. Additionally, significant differences were observed in the macular VD and PD areas within the inferior-inner, inferior-outer, superior-outer, superior-inner, and nasal-outer quadrants among PwMS with ON compared to PwNMOSD also experiencing ON [34].

A study by Rogaczewska et al. on 40 PwMS and 13 PwNMOSD identified the macular and peripapillary neurovascular alterations through OCT-A to distinguish between NMOSD and MS eyes; it was found that in PwNMOSD with ON, reduced RPC vessel density was observed in the superior and inferior quadrants compared to PwMS with ON. Additionally, the RPC quadrant ratios (superior to temporal, inferior to nasal, and inferior to temporal) were significantly lower in PwNMOSD. In PwMS without ON, the RPC vessel density was significantly decreased in the temporal quadrant compared to PwNMOSD without ON. Additionally, the superior-to-temporal and nasal-to-temporal ratios were lower in PwNMOSD. The optimal parameters for differentiating NMOSD from MS were the inferior-to-nasal and inferior-to-temporal ratios in cases of ON and the superior-to-temporal and nasal-to-temporal ratios in non-ON cases [31].

In another study with a similar population, they also revealed that in PwNMOSD with ON, the sectoral vessel density was significantly reduced in the inferior, superior nasal, and nasal superior sectors compared to PwMS with ON. Additionally, PwMS without ON exhibited lower RPC density in the inferior nasal and temporal superior sectors compared to PwNMOSD without ON [32].

In a study conducted by Lee et al., which included eyes with ON from 23 PwMS and 37 PwNMOSD, it was found that the PwNMOSD exhibited lower VD in both the superficial radial capillary plexus (SRCP) in average, superior, and inferior segments and RPC in average, superior, inferior, nasal, and temporal segments compared to PwMS. However, vessel density in the deep radial capillary plexus (DRCP) did not show significant differences between the two groups [22].

A study by Aly et al. involving 21 PwMS and 16 PwNMOSD to investigate retinal vascular changes specific to each condition identified no vascular differences between the ON eyes of PwMS and PwNMOSD. Moreover, in eyes lacking a history of ON, the study found no discrepancies in the densities of the superficial vascular complex (SVC) and deep vascular complex (DVC) between the two patient groups [35].

Tiftikcioglu et al. conducted a study using OCT-A to analyze the retinal superficial peripapillary and macular capillary networks in 14 PwMS and 9 PwNMOSD with or without ON history, revealing that PwNMOSD with ON had significantly reduced both peripapillary and macular VD compared to PwMS with ON. Additionally, seronegative PwNMOSD with ON exhibited lower peripapillary VD than PwMS with ON. PwNMOSD with ON also demonstrated a smaller FAZ area and perimeter than PwMS with ON. However, there were no differences in peripapillary and macular VD between eyes without a history of ON between PwMS and PwNMOSD. Notably, PwNMOSD without ON had a smaller FAZ area compared to PwMS without ON [7].

The summary of the major findings of the included studies is presented in Table 2.

**Table 2:** Summary of key findings from included studies comparing OCT-A parameters in MS and NMOSD

Study	Sample Size (MS/NMOSD)	ON Status	OCT-A Device/ Software	Key OCT-a Parameters	Main Findings
Liu et al. (2024) [21, 34]	83 / 91	Mixed ON & non-ON	Zeiss AngioPlex	VD, PD	PwMS had higher macular VD/PD in most quadrants than PwNMOSD, especially in ON eyes.
Rogaczewska et al. (2021) [31, 32]	40 / 13	ON & non-ON groups	Optovue AngioVue	RPC, SCP, DCP	PwNMOSD with ON had reduced RPC density and quadrant ratios; FAZ smaller in NMOSD.
Lee et al. (2021) [22]	23 / 37	ON only	Zeiss Topcon OCT instrument	SRCP, DRCP, RPC	PwNMOSD had lower VD in SRCP and RPC, no significant difference in DRCP.
Aly et al. (2022) [35]	21 / 16	ON & non-ON	Optovue AngioVue	SVC, DVC, FAZ	No vascular differences between ON eyes; FAZ smaller in NMOSD without ON.
Tiftikcioglu et al. (2022) [7]	14 / 9	Mixed	Zeiss AngioPlex	VD, FAZ	PwNMOSD with ON showed lower peripapillary VD and smaller FAZ; also, in non-ON eyes.

DCP: deep capillary plexus, DRCP: deep radial capillary plexus, FAZ: foveal avascular zone, OCT-A: optical coherence tomography angiography, ON: optic neuritis, PD: perfusion density, PwMS: people with multiple sclerosis, PwNMOSD: people with neuromyelitis optica spectrum disorder, RPC: radial peripapillary capillary, SCP: superficial capillary plexus, SRCP: superficial radial capillary plexus, SVC: superficial vascular complex, VD: vessel density

### Risk of bias assessment

Table 1 summarizes the ROB of the included studies assessed using the NOS. The average (SD) rating was 6.6 (SD 1.1), indicating a moderate to high overall quality level.

## Discussion

The findings from the included studies demonstrated significant retinal vascular differences between PwMS and PwNMOSD, particularly in those with a history of ON. PwNMOSD consistently exhibited lower vessel densities across various retinal regions, including the peripapillary and macular areas, reduced RPC density, and smaller FAZ areas compared to PwMS. However, in eyes without a history of ON, retinal vascular parameters show minimal or no differences, indicating that ON may be a key factor in differentiating these disorders using OCT-A metrics. Furthermore, some studies revealed that the inferior and superior quadrants of the peripapillary region may show measurable differences between MS and NMOSD groups, suggesting a possible role in aiding disease discrimination.

Limited studies have explored retinal vascular alterations between PwMS and PwNMOSD. Optic nerve head perfusion measurements in ON have demonstrated diagnostic value by reflecting the severity of optic nerve damage [36]. Some studies suggested that intraocular vascular changes in MS or NMOSD may result from secondary vascular regression caused by axonal decline [22], which reduces retinal metabolic activity and subsequently lowers oxygen and nutrient demand [12, 37]. However, others indicated intraretinal perfusion changes in MS or NMOSD may also result from primary vascular dysfunction [12]. The inconsistent findings of previous studies have led to a lack of consensus on whether microvascular changes in MS [12, 36, 38, 39] or NMOSD [14, 40] result from primary retinal vasculopathy, secondary vascular regression following optic atrophy, or vascular damage associated with ON-related acute optic nerve inflammation.

Prior ophthalmic research has consistently demonstrated greater functional and structural impairments in NMOSD than in MS, regardless of the presence [41–43] or absence [44] of ON episodes. The VD in the SRCP and RPC segments is significantly reduced in PwNMOSD compared to PwMS, potentially reflecting the greater severity of optic nerve damage in NMOSD [22]. Increased axonal injury in NMOSD may lead to a more pronounced decrease in the metabolic demand for oxygen and nutrients, thereby accounting for a greater reduction in perfusion [22]. The reduction in RPC vessel density, particularly within the superior and inferior quadrants, was more pronounced in both ON and non-ON eyes of PwNMOSD patients when compared to PwMS [31]. The predominant emergence of major retinal vessels from the optic disc at its superior and inferior margins corresponds with prior evidence demonstrating retinal vascular attenuation in NMOSD-affected eyes [31, 45]. Additionally, the difference in FAZ size between NMOSD and MS eyes may be due to greater or persistent foveal inflammation in NMOSD, reflecting active disease, unlike MS, where inflammation is considered more self-limiting over time [7].

The variations in vessel densities between the two disorders may result from distinct pathophysiological mechanisms underlying primary vasculopathy in MS and NMOSD [22]. The peripapillary retinal vessels are encapsulated by the end-foot processes of macroglial cells, namely astrocytes and Müller cells, which play a critical role in forming and maintaining the inner blood-retina barrier [46]. Perivascular inflammation, characterized by microvascular impairment and prominent vascular changes, is a shared pathological feature of MS [47] and NMOSD [48]. However, unlike MS, where glial fibrillary acidic protein (GFAP) expression in lesions is typically upregulated [49], NMOSD lesions exhibit a loss of GFAP-positive astrocytes [50, 51]. As a cytoskeletal protein, GFAP plays a crucial role in astrocyte structure and motility [49], with astrocytes acting as intermediaries between neuronal activity and vascular responses [52–54]. The end-foot processes of astrocytes are highly enriched with AQP4, a water channel targeted by AQP4-IgG, under inflammatory conditions in NMOSD [46]. Given that these end-foot processes are the primary target of AQP4 antibodies [55–58], NMOSD is associated with astrocyte dysfunction and impaired neurovascular coupling [59–61], likely as a result of chronic autoimmune inflammation directed at retinal astrocytes [62]. These pathophysiological differences between MS and NMOSD may underlie the distinct alterations observed in retinal microvascular structures [22].

Subclinical retinal damage may occur partially independent of acute inflammatory episodes affecting the optic nerve [63, 64]. PwNMOSD may exhibit subclinical parafoveal retinal vessel loss, which is associated with astrocyte damage and reduced visual function [35]. Furthermore, decreased peripapillary and foveal vessel density is observed in NMOSD, irrespective of prior ON episodes [21]. In PwNMOSD with a history of ON, a reduction in retinal vascular network density may precede the onset of ON and peripapillary retinal nerve fiber layer (pRNFL) atrophy [20]. Regarding MS, microvascular changes have been identified in people with early-stage MS, clinically isolated syndrome, and various MS subtypes [12, 65, 66]. OCT-A studies have demonstrated that blood perfusion in the optic nerve head, but not in the parafovea, is reduced in MS eyes with a history of ON compared to MS eyes without ON [36]. Furthermore, a reduction in optic nerve head perfusion has also been observed in MS eyes without a history of ON [39]. Overall, preliminary findings from some studies suggest that OCTA may complement OCT in distinguishing MS from NMOSD [34], but further validation is necessary. Liu et al. suggested that combining OCTA with OCT may offer improved diagnostic insights [34], though this needs confirmation through larger, prospective studies.

### Limitations

This review was carried out with rigorous measures to ensure reliability and validity while minimizing bias, including applying well-defined inclusion and exclusion criteria, systematic search methods, detailed data extraction, evidence grading, and a thorough evaluation of all relevant publications. Nonetheless, as with any systematic review, certain limitations remain inherent. Research specifically exploring OCT-A indices to differentiate between MS and NMOSD is limited, with significant variability in methodology and analysis across included studies. Differences in OCT-A devices and protocols, seropositivity status of PwNMOSD, history of ON, and outcome measures created challenges for meaningful meta-analysis. Additionally, the predominance of cross-sectional study designs restricts the capacity to determine causation or the directionality of associations [67]. The small number of PwMS and PwNMOSD in the included studies represents a major limitation, undermining the validity and restricting the generalizability of the findings. OCT-A vessel density measurements quantify angiographic signals based on motion; however, factors such as blood flow velocity, vascular morphology, and endothelial barrier integrity can influence perfusion measurements among the included studies. The OCT-A measurements in the included studies encompassed large, small, and combination blood vessels, potentially increasing the risk of quantification bias. Moreover, another limitation of this review is the heterogeneity in the regions of interest (ROIs) analyzed across the included studies. Differences in OCT-A scanning protocols, fields of view, and segmentation settings across devices could have influenced the quantitative outcomes and comparability of vessel density measurements. Additionally, OCT-A imaging is susceptible to motion and segmentation artefacts, which can further compromise measurement reliability and consistency across studies. The measurements were acquired using different OCT-A devices, leading to variations in the peripapillary annulus diameter used for retinal vessel density calculations. The discrepancies in the analyzed areas may challenge the direct comparability of the results of the studies. PwNMOSD among the included studies consisted of AQP4-IgG seropositive or seronegative PwNMOSD or a combination of them. As all the included studies were retrospective, no analysis was performed to assess the impact of treatment strategies on the outcomes. Therefore, prospective cohort studies are necessary to validate the findings and further investigate alterations in vessel density.

## Conclusion

This review identified differences in OCT-A indices between PwMS and PwNMOSD, with PwNMOSD exhibiting lower vessel densities in the peripapillary and macular regions and smaller FAZ areas compared to PwMS. While OCTA parameters appear to differ between MS and NMOSD in certain contexts, current evidence is insufficient to establish their definitive diagnostic utility. These preliminary findings suggested that OCTA may provide complementary information to existing clinical, radiological, and serological tools. However, larger, standardized, and longitudinal studies are required to determine the diagnostic and prognostic relevance of OCTA metrics in clinical practice.

## Electronic supplementary material

Below is the link to the electronic supplementary material.

Supplementary Material 1

## Data Availability

The data used to support the findings of this study are included within the article.

## Abbreviations

AQP4-ab: Autoantibodies targeting aquaporin-4
CMS: Central nervous system
DRCP: Deep radial capillary plexus
FAZ: Foveal avascular zone
GCL: Ganglion cell layer
MOGAD: Myelin oligodendrocyte glycoprotein antibody-associated disease
MS: Multiple sclerosis
NMOSD: Neuromyelitis optica spectrum disorder
NOS: Newcastle–Ottawa scale
OCT: Optical coherence tomography
OCT-A: Optical coherence tomography angiography
ON: Optic neuritis
PD: Perfusion density
PwMS: People with multiple sclerosis
PwNMOSD: People with neuromyelitis optica spectrum disorder
RNFL: Retinal nerve fiber layer
RCP: Retinal capillary plexus
RPC: Radial peripapillary capillary
VD: Vessel density
