# Supplementary material for: Optical coherence tomography angiography biomarkers in multiple sclerosis and neuromyelitis optica spectrum disorders: a systematic review
Source: Int J Retina Vitreous. 2025 Jun 23;11:71. doi: 10.1186/s40942-025-00698-x (PMC12183867; doi:10.1186/s40942-025-00698-x)
Supplement: Supplementary file 1 — Supplementary Material 1 [file 40942_2025_698_MOESM1_ESM.docx]

**Supplementary Material**

**Table S1.** The search strategy for each database.

| Syntax | Results |
| --- | --- |
| Pubmed/MEDLINE | 544 |
| ((sclerosis AND multiple) OR (sclerosis AND disseminated) OR "disseminated sclerosis" OR "multiple sclerosis" OR "acute fulminating")  AND  (“Neuromyelitis Optica Spectrum Disorder” OR “Neuromyelitis Optica Spectrum Disorders” OR “Neuromyelitis Optica” OR “NMO Spectrum Disorder” OR “NMO Spectrum Disorders” OR “Neuromyelitis Optica (NMO) Spectrum Disorder” OR “Devic Neuromyelitis Optica” OR “Devic Neuromyelitis Opticas” OR (“Neuromyelitis Optica” AND Devic) OR (“Neuromyelitis Opticas” AND Devic) OR “Devic's Disease” OR “Devics Disease” OR (Disease AND Devic's) OR “Devic Disease” OR (Disease AND Devic) OR “Devic Syndrome” OR (Syndrome AND Devic) OR “Devic's Syndrome” OR “Devics Syndrome” OR (Syndrome AND Devic's) OR “Devic's Neuromyelitis Optica” OR “Devics Neuromyelitis Optica” OR (“Neuromyelitis Optica” AND Devic's) OR “Neuromyelitis Optica (NMO) Spectrum Disorders”)  AND  (“Optical Coherence Tomography” OR (“Coherence Tomography” AND Optical) OR (Tomography AND “Optical Coherence”) OR “OCT Tomography” OR (Tomography AND “OCT”) OR “Optical Coherence Tomography Angiography” OR OCT OR OCTA) |  |
| Scopus | 129 |
| TITLE-ABS((sclerosis AND multiple) OR (sclerosis AND disseminated) OR "disseminated sclerosis" OR "multiple sclerosis" OR "acute fulminating")  AND  TITLE-ABS(“Neuromyelitis Optica Spectrum Disorder” OR “Neuromyelitis Optica Spectrum Disorders” OR “Neuromyelitis Optica” OR “NMO Spectrum Disorder” OR “NMO Spectrum Disorders” OR “Neuromyelitis Optica (NMO) Spectrum Disorder” OR “Devic Neuromyelitis Optica” OR “Devic Neuromyelitis Opticas” OR (“Neuromyelitis Optica” AND Devic) OR (“Neuromyelitis Opticas” AND Devic) OR “Devic's Disease” OR “Devics Disease” OR (Disease AND Devic's) OR “Devic Disease” OR (Disease AND Devic) OR “Devic Syndrome” OR (Syndrome AND Devic) OR “Devic's Syndrome” OR “Devics Syndrome” OR (Syndrome AND Devic's) OR “Devic's Neuromyelitis Optica” OR “Devics Neuromyelitis Optica” OR (“Neuromyelitis Optica” AND Devic's) OR “Neuromyelitis Optica (NMO) Spectrum Disorders”)  AND  TITLE-ABS(“Optical Coherence Tomography” OR (“Coherence Tomography” AND Optical) OR (Tomography AND “Optical Coherence”) OR “OCT Tomography” OR (Tomography AND “OCT”) OR “Optical Coherence Tomography Angiography” OR OCT OR OCTA) |  |
| Web of Science | 288 |
| TS=((sclerosis AND multiple) OR (sclerosis AND disseminated) OR "disseminated sclerosis" OR "multiple sclerosis" OR "acute fulminating")  AND  TS=(“Neuromyelitis Optica Spectrum Disorder” OR “Neuromyelitis Optica Spectrum Disorders” OR “Neuromyelitis Optica” OR “NMO Spectrum Disorder” OR “NMO Spectrum Disorders” OR “Neuromyelitis Optica (NMO) Spectrum Disorder” OR “Devic Neuromyelitis Optica” OR “Devic Neuromyelitis Opticas” OR (“Neuromyelitis Optica” AND Devic) OR (“Neuromyelitis Opticas” AND Devic) OR “Devic's Disease” OR “Devics Disease” OR (Disease AND Devic's) OR “Devic Disease” OR (Disease AND Devic) OR “Devic Syndrome” OR (Syndrome AND Devic) OR “Devic's Syndrome” OR “Devics Syndrome” OR (Syndrome AND Devic's) OR “Devic's Neuromyelitis Optica” OR “Devics Neuromyelitis Optica” OR (“Neuromyelitis Optica” AND Devic's) OR “Neuromyelitis Optica (NMO) Spectrum Disorders”)  AND  TS=(“Optical Coherence Tomography” OR (“Coherence Tomography” AND Optical) OR (Tomography AND “Optical Coherence”) OR “OCT Tomography” OR (Tomography AND “OCT”) OR “Optical Coherence Tomography Angiography” OR OCT OR OCTA) |  |
| Embase | 250 |
| ((sclerosis:ab,ti AND multiple:ab,ti) OR (sclerosis:ab,ti AND disseminated:ab,ti) OR ‘disseminated sclerosis’:ab,ti OR ‘multiple sclerosis’:ab,ti OR ‘acute fulminating’:ab,ti)  AND  (‘Neuromyelitis Optica Spectrum Disorder’:ab,ti OR ‘Neuromyelitis Optica Spectrum Disorders’:ab,ti OR ‘Neuromyelitis Optica’:ab,ti OR ‘NMO Spectrum Disorder’:ab,ti OR ‘NMO Spectrum Disorders’:ab,ti OR ‘Neuromyelitis Optica (NMO) Spectrum Disorder’:ab,ti OR ‘Devic Neuromyelitis Optica’:ab,ti OR ‘Devic Neuromyelitis Opticas’:ab,ti OR (‘Neuromyelitis Optica’:ab,ti AND Devic:ab,ti) OR (‘Neuromyelitis Opticas’:ab,ti AND Devic:ab,ti) OR ‘Devics Disease’:ab,ti OR ‘Devic Disease’:ab,ti OR (Disease:ab,ti AND Devic:ab,ti) OR ‘Devic Syndrome’:ab,ti OR (Syndrome:ab,ti AND Devic:ab,ti) OR ‘Devics Syndrome’:ab,ti OR ‘Devics Neuromyelitis Optica’:ab,ti OR ‘Neuromyelitis Optica (NMO) Spectrum Disorders’:ab,ti)  AND  (‘Optical Coherence Tomography’:ab,ti OR (‘Coherence Tomography’:ab,ti AND Optical:ab,ti) OR (Tomography:ab,ti AND ‘Optical Coherence’:ab,ti) OR ‘OCT Tomography’:ab,ti OR (Tomography:ab,ti AND ‘OCT’:ab,ti) OR ‘Optical Coherence Tomography Angiography’:ab,ti OR OCT:ab,ti OR OCTA:ab,ti) |  |
